# Frequency of Red Blood Cell Alloimmunization in Patients with Sickle Cell Disease in Palestine

**DOI:** 10.1155/2018/5356245

**Published:** 2018-06-06

**Authors:** Fekri Samarah, Mahmoud A. Srour, Dirgham Yaseen, Kamal Dumaidi

**Affiliations:** ^1^Department of Medical Technology, Faculty of Allied Health Sciences, Arab American University in Jenin, State of Palestine; ^2^Department of Biology and Biochemistry, Faculty of Science, Birzeit University, Birzeit, State of Palestine; ^3^Rafidia Governmental Hospital, Ministry of Health, Nablus, State of Palestine

## Abstract

**Background:**

Transfusion of red blood cells (RBC) is an essential therapeutic tool in sickle cell disease (SCD). Repeated RBC transfusions can cause alloimmunization which causes difficulty in cross-matching and finding compatible blood for transfusions. This study aimed to investigate the frequency of RBC alloimmunization and related risk factors among Palestinian SCD patients.

**Materials and Methods:**

A multicenter cross-sectional study on 116 previously transfused SCD patients from three centers in West Bank, Palestine. Demographic, medical data and history of transfusion were recorded. Blood samples were collected from transfused consenting SCD patients. Gel card method was used for antibody screening and identification. In all patients, autocontrol and direct antiglobulin (DAT) test were performed using polyspecific (anti-IgG + C3d) anti-human globulin (AHG) gel cards for the detection of autoantibodies.

**Results:**

Of the SCD patients, 62 (53.4%) patients were HbSS and 54 (46.6%) patients were sickle *β*-thalassemia (S/*β*-thal). There were 53 (45.7%) females and 63 (54.3%) males. Mean age was 18.8 years (range 3-53 years). The frequency of RBC alloimmunization among SCD patients was 7.76%, with anti-K showing the highest frequency (33.3%) followed by anti-E (22.2%), anti-D (11.1%), anti-C (11.1%), and anti-c (11.1%). All reported IgG alloantibodies were directed against antigens in the Rh (66.7%) and Kell (33.3%) systems. Older ages of patients, increased number of blood units transfused, and splenectomy were the commonest risk factors for alloimmunization in our study.

**Conclusions:**

RBC alloimmunization rate among Palestinian SCD patients is low compared to neighboring countries and countries all over the world but still warrants more attention. Phenotyping of donors/recipients' RBC for Rh antigens and K_1_ (partial phenotype matching) before their first transfusion may reduce the incidence of alloimmunization.

## 1. Introduction

Of all inherited diseases of human, sickle cell disease (SCD) is remarkable due to its pathology and disease complications and challenges it provides compared to other diseases. Factors that contribute to the unique character of SCD include its wide geographic distribution, chronicity, and poor response to therapy. SCD results from genetic mutations in the *β*-globin gene. The control of SCD, as well as its cure, still eludes physicians, research workers, and social scientists [1]. In Palestine, *β*-thalassemia and SCD are among the most common inherited abnormalities of hemoglobin synthesis. The prevalence of sickle cell trait in West Bank region of Palestine is estimated to be 1.2% based on the documentation of 116 cases of homozygote sickle cell anemia (HbSS) in the West Bank region [2] with a population of three million [3]. While the prevalence of *β*-thalassemia trait in West Bank region is 3.5% [4]. Analysis of the *β*^S^-globin gene cluster haplotypes in a cohort of Palestinian patients homozygotes, for HbS [*β*6(A3)Glu→Val, GAG>GTG], revealed that these patients had low levels of HbF and severe clinical course, which contributes to a shortened lifespan. The *β*^S^-mutation present in Palestine has been traced back to the Benin region and has been probably brought to Palestine along the slave trade routes [5]. Complications of SCD are attributed mainly to chronic hemolysis and painful crises due to vasooclusions, ischemia, and inflammations [6]. SCD includes all genotypes of homozygous HbSS as well as cases of compound heterozygotes with other hemoglobinopathies such as *β*-thalassemia (*β*^0^ or *β*^+^), with varying degrees of severity influenced by the amount of HbA production [7]. The last three decades have witnessed improvements in the management and treatment of SCD [8]. Hydroxyurea administration has improved SCD patient outcomes and morbidity [9]. Packed red blood cell (RBC) transfusion is an essential therapeutic component in the management of SCD, in both acute and chronic episodes like splenic sequestration crisis, acute chest syndrome (ACS), and many others [10]. Blood transfusion improves the oxygen-carrying capacity of patient's blood by reducing the level of HbS and increasing its hemoglobin concentration [11]. However, alloimmunization to RBC group antigens is a major complication of allogeneic blood transfusion and generally presents significant challenges in the management of SCD patients [12]. The frequency of RBC alloimmunization in SCD patients was reported to range from 7 to 47% [13]. In the United States, the Cooperative Study of Sickle Cell Disease revealed that more than 50% of alloimmunized SCD patients showed multiple antibodies [14]. The high frequency of alloimmunization in patients with SCD is not clearly understood. One of the most important mechanisms responsible for the development of alloimmunization is the RBC genetic disparity between donors and recipients due to racial differences [15]. Additional risk factors may include older age and sex, increased number and timing of blood transfusions, use of nonleukoreduced RBC, pregnancy, use of long-term stored blood products, patient's diagnosis, and genetic factors [9, 10, 13–17]. Alloimmunization has been associated with increased morbidity in SCD, due to the difficulty in cross-matching to find compatible blood for future transfusion, autoantibody formation, hemolysis, and delayed transfusion reactions [15, 18]. The most frequently reported alloantibodies identified in SCD are those directed against antigens of the Rh and Kell systems, followed by the Kidd and Duffy systems [9, 10, 13–16, 19]. Blood transfusion in Palestine takes place in both public and private hospitals, which are all governed by the guidelines and directions of the Palestinian Ministry of Health (MOH) on blood bank issues. To the best of our knowledge, no similar previous studies are available in Palestine. Therefore the aim of this study was to determine the frequency of RBC alloimmunization and the related risk factors among Palestinian SCD patients.

## 2. Materials and Methods

### 2.1. Study Design and Population

This was a multicenter cross-sectional study. SCD patients from three major governmental hospitals in Nablus, Jenin, and Tulkarem in West Bank region were included in this study. A total of 116 SCD patients were recruited between January and December 2017. Of these, 62 patients have sickle cell anemia (HbSS) and 54 patients have sickle *β*-thalassemia. The inclusion criteria included all transfusion dependent SCD patients as well as those that have had a history of at least two transfusions of ABO- and RhD-matched RBC during their lifespan.

The study was approved by the Palestinian MOH and the principles of Helsinki Declaration were implemented. Written informed consent was obtained from patients or their guardians in case of minors. A special questionnaire was used to collect demographic and medical data including patient's age, sex, age on first RBC transfusion, ABO and Rh blood group, hemoglobin concentration, history of splenectomy, number of blood units transfused, transfusion reactions, frequency, and specificity of alloantibodies by direct interview of patients or their guardians and from medical files. Clinical files and transfusion records were analyzed in all SCD patients for the presence of alloantibodies in SCD patients.

### 2.2. Laboratory Investigations

The diagnosis of SCD patients included in this study was based on clinical (stated in their medical records) and laboratory investigation including hemoglobin electrophoresis as well as B-globin gene mutation analysis. Complete blood count (CBC) including hemoglobin concentration and mean cell volume (MCV) was measured using Nihon Kohden (MEK-6510K) (Diamond Diagnostics, Japan) cell counter. Sickle cell phenotypes were diagnosed by conventional electrophoresis methods (cellulose acetate at alkaline and acid pH) [20]. Homozygosity for the *β*^S^-mutations was determined by RFLP-PCR using the restriction enzyme DdeI as described earlier [21]. S/*β*^0^-thal resembles HbSS clinically, but here S/*β*^0^-thal cases were differentiated from HbSS by the presence of heterozygous *β*^S^-mutations, elevated HbA2 concentrations, low MCV, and family history [7].

### 2.3. Alloantibody Testing

SCD patients enrolled in this study were transfused with ABO- and RhD-matched packed RBC at the three healthcare centers included in this study. This was the transfusion policy adopted at all government hospitals in West Bank until 2014. From 2014 and on, a new pretransfusion strategy for patients was applied including antibody screening and identification tests.

In this study, pretransfusion blood samples were obtained from each patient in two separate tubes, K2-EDTA tube for direct globulin test (DAT) and autocontrol (for detection of autoantibodies) and the second tube without anticoagulant (plain) (for cross-matching, antibody screening, and identification). All immunohematological tests were performed using the gel card method (Diamed ID, Switzerland), according to the manufacturer's instructions. Pretransfusion blood samples from all SCD patients were tested for the presence of alloantibodies by gel technology using commercial 3-cell panel (Diacell, Diamed ID, Switzerland), with polyspecific (anti-IgG + C3d) anti-human globulin (AHG) and homozygous expression of the antigens. All samples testing positive for alloantibody screening were further tested to identify the antibody specificity using an extended panel of 11 cells (Diacell, Diamed ID, Switzerland). Autocontrol was performed with each sample to detect autoantibodies (patient's RBC with patient's serum). Direct antiglobulin test (DAT) was also performed for each sample. In cases with a positive DAT, elution and adsorption methods were employed. Specific reagents to detect IgG, IgM, or complement were carried out using standard laboratory techniques. The alloimmunization state was ascertained by the presence of antibodies to one or more RBC antigens.

### 2.4. Statistical Analysis

Descriptive statistics and Chi-square analysis were performed using IBM SPSS statistics (version 23). Data were recorded as mean ±SD. A p value less than 0.05 was considered statistically significant.

## 3. Results

A total of 116 SCD patients, attending three thalassemia governmental units in Palestine (Nablus, Jenin, and Tulkarem) were included in the study. The group of SCD patients included in this study was phenotypically distributed as follows: 62 patients (53.4%) with HbSS and 54 (46.6%) patients with S/*β*-thal. Of the S/*β*-thal patients, 39 (72.2%) were S/*β*^0^-thal and 15 (27.8%) were S/*β*^+^-thal. The study population included 53 (45.7%) females and 63 (54.3%) males. Mean age was 18.8 ± 9.83 (range of 3-53 years) for all patients. Nine patients (7.76%) developed alloantibodies and 107 (92.24%) did not. The mean age of the alloimmunized and nonalloimmunized participants was 24 (range, 8-48) years and 18.4 (range, 3-53) years, respectively (Table 1). Our data indicated that there was no association between gender and the rate of alloimmunization (*P*=0.73). The rates of alloimmunization among female and male SCD patients were 9.4% (5/53) and 6.3% (4/63), respectively (Table 1).

To investigate the association between age and risk of alloimmunization, patients were categorized into two age groups. As shown in Table 1, there was a statistically significant association between age and rate of alloimmunization (*P*=0.001). Most SCD patients are young patients, where children (<14 years) accounted for 62.9% (n=73/116) of all patients. Most alloimmunized SCD patients (8 out of 9) were adult patients, while only one alloimmunized patient was observed among the child SCD patients. Thus our data indicated that the rate of alloimmunization increased with increasing age. Alloimmunization was more frequent in S/*β*-thal (9.26%) than in HbSS (6.45%) patients but was statistically insignificant (*P*=0.73). Splenectomy was more common in alloimmunized than nonalloimmunized patients and the difference was statistically significant (*P*=0.009).

In our study, the patients were transfused with a total of 3441 units of blood (mean= 29.7 ± 19.1; range 4-98) (Table 1). A total of 40 SCD patients were on the chronic transfusion regimen, 15 patients with homozygous HbSS and 25 patients with HbS/B-thal. All the alloimmunized SCD patients were on chronic transfusion regimen. Our data indicated that there was a statistically significant difference in the number of blood transfusion units between the alloimmunized group compared to nonalloimmunized group (*P*=0.019), with the alloimmunized group having a higher number of transfusions (Table 1).

Data presented in Table 2 shows the type and frequency of alloantibodies reported in our study. IgG alloantibodies against RBC antigens were detected in nine (7.76%) of the 116 SCD patients who received blood transfusions. All reported IgG alloantibodies were directed against antigens in the Rh (66.7%) and Kell (33.3%) systems. Alloantibody against K had the highest frequency (33.3%) followed by E (22.2%), D (11.1%), C (11.1%), and c (11.1%), respectively. One case (0.86%) of 116 SCD patients in the nonalloimmunized patients developed autoantibody as revealed by positive polyclonal autocontrol and direct antiglobulin tests.

Table 3 shows the profile of patients who developed RBC alloantibodies in our study. All the RBC alloimmunized SCD patients were on chronic blood transfusion and received a higher rate of transfusion (53.3 ± 22.2 RBC units). The most frequent indications for blood transfusion in all patients were anemia and vasooclussive crisis. Transfusion reactions ranged between immediate hemolytic transfusion reaction (IHTR) in one patient, asymptomatic in 3 patients, and delayed hemolytic transfusion reactions (DHTR) in the rest.

## 4. Discussion

The high prevalence of the *β*^S^ mutation in Africa, the Middle East, India, and parts of the Mediterranean has a relation to the selective protective effect of HbS against malaria. Its spread to other regions of the world may be due to the slave trade and/or population migration [7]. RBC transfusion is an essential therapeutic component in the management of acute and chronic complications in SCD patients, where 90% of patients will have been transfused at least once in their life [22]. Despite the importance of RBC transfusions in improving SCD patients' outcomes and morbidity, alloimmunization to RBC group antigens is one of the major adverse effects of allogeneic blood transfusion [12]. Alloimmunization is a multifactorial process, but at least three elements are most important: the antigenic differences between the donor and recipient, the immune status of the recipient, and the immunomodulatory effect of the allogeneic blood transfusion on the immune system of the recipient [15, 18]. There are no published data on the frequency of alloimmunization and related risk factors among Palestinian SCD patients. Thus, this study aimed to investigate the frequency of alloimmunization among these patients. The rate of alloimmunization observed in the present study was 7.76%, which is lower than that reported in many studies of RBC alloimmunization in SCD patients [10, 12–15, 19, 23–25]. Other studies present lower rates of RBC alloimmunization [16, 26–28]. These differences in the rate of RBC alloimmunization among SCD patients support the importance of ethnic/genetic differences between patients and donors. The low rate of alloimmunization in the present study (7.76%) may be due to the high phenotypic compatibility between SCD patients and blood donors although donors were not related to the patients, but they were all Palestinians. Although the cost of antigen matching is high, further studies are needed to investigate the influence of this factor on the rate of alloimmunization. Another factor that could contribute to the relatively low rate is that SCD patients are not checked for RBC alloantibodies after each transfusion which may lead to missing the detection of transitory alloantibodies. In the healthcare centers included in this study, antibody screening is performed at the pretransfusion stage and is not retested routinely unless a new transfusion is ordered. Our results showed that the majority of SCD patients had a single alloantibody rather than multiple alloantibodies. Of all alloantibodies detected, anti-K (33.3%) was the most frequent alloantibody followed by anti-E (22.2%), anti-D (11.1%), anti-C (11.1%), and anti-c (11.1%), respectively. Given that both Rh and Kell systems have highly immunogenic antigens and phenotype-matched RBC was not performed for these antigens except D, the prevalence of alloantibodies of both systems among our study subjects was similar to previous reports [9, 10, 13–16, 19]. Donor RBC phenotyping for Rh (D, C, E, c, and e) and K_1_ (partial phenotype matching) is necessary to avoid alloimmunization and stop unwanted clinical consequences in SCD patients [25]. Castro et al. reported a drop in the rate of alloimmunization from 3% to 0.5% when SCD patients were transfused only with phenotype-matched RBC [29]. Anti-D was found in two SCD patients (22.2%) where in one case it was a single alloantibody and in the second case it was in combination with anti-C, a frequency higher than that reported in most studies [14–16, 19]. The two patients with anti-D were males, the first patient was 8 years old and the other patient with the anti-D and anti-C aged 54 years. The two patients had been typed as RhD+ by serology, and thus further molecular [30] and serological studies must be conducted in these patients to unequivocally establish their RhD status. Indeed, Natukunda et al. who performed genotyping for RhD in SCD patient's alloimmunized with anti-D found that such patients had either partial D or D pseudogenes [16]. These findings are encouraging to improve the standards of blood bank services in our centers. The current study showed that there was no association between gender and the rate of alloimmunization with 4 (6.3%) male patients and 5 (9.4%) female patients having alloantibodies, respectively (p=0.73) (Table 1). Only a few reports showed that gender was not a significant factor for RBC alloimmunization [13, 14]. Other studies reported a significant association between gender and RBC alloimmunization [25, 31]. Since none of the female subjects in our study get married and the majority of patients were predominantly in the pediatric age, gender was not a risk factor. Alloimmunization rate was not significantly depending on the type of sickle cell disease since HbSS and S/*β*-thal subjects showed alloimmunization rate with frequencies of 6.45% and 9.26%, respectively (*P*=0.73). Age has been associated with the risk of RBC alloimmunization in SCD [14, 15]. By analysis of the frequency of alloimmunization in different age groups, our data revealed that the highest rate (55.6%) was among patients with older age (> 20 years). These patients received multiple blood units for several years and thus were more exposed to allogeneic RBC in their life [14, 32]. The starting time of blood transfusion in our study population was difficult to identify because of the poor filing system. Rosse et al. reported that SCD children who were first transfused at age of 10 years and older had a higher rate of alloantibodies compared to those who were transfused before that age [14]. Other authors had reported similar results [32, 33]. Factors that modify the low rate of alloimmunization in SCD children include immune tolerance, absence of pregnancy, and lower frequency of blood transfusions [18]. Thus, starting transfusion at early age in pediatric SCD patients may provide protection against RBC alloantibodies because of immune tolerance induction. This study shows that the risk of alloimmunization increases with the number of blood units transfused. This finding is in agreement with previous reports that RBC allosensitization is more likely in patients with increased frequency of blood transfusion [34–36]. The alloimmunized SCD patients in Palestine received a high rate of transfusion (53.3 ± 22.2; range 20-98 RBC units). In the current study, twenty-five SCD patients underwent splenectomy of whom 7 (28%) had alloantibodies. Our findings revealed a significant association between splenectomy and rate of alloimmunization (*P*=0.009), since spleen plays an important role in red cell turnover. Additionally, our findings revealed that splenectomy is a risk factor for RBC alloimmunization and this is consistent with the findings of earlier reports [37, 38], while other studies revealed no significant association [39]. In the present study, one patient (0.86%) in the nonalloimmunized group developed an autoantibody. The pathogenesis of autoantibodies following transfusion in SCD patients is not well understood. Aygun et al. reported an association between IgG autoantibodies and clinically significant hemolysis in 8% of pediatrics and 9.7% of adults [40], while others found no clinical association with hemolysis [41].

## 5. Conclusion

This was the first report of the frequency of RBC alloimmunization and the related risk factors among SCD patients in Palestine. All alloantibodies identified among our study population were clinically significant and are mostly against Rh and Kell blood group systems. Thus we recommend that donors of RBC, as well as recipients' of RBC, should be phenotyped for Rh (D, C, E, c, and e) and K_1_ (partial phenotype matching) before the first transfusion to avoid alloimmunization in SCD patients. Older age of patients, increase in number of blood units transfused, and splenectomy were the common risk factors for alloimmunization among our study population.

## Figures and Tables

**Table 1:** SCD patients' demographic and transfusion characteristics in Palestine.

**Variable**	**SCD alloimmunized**	**SCD nonimmunized**	**Total**	***P*-value**
n (%)	9 (7.76%)	107 (92.24%)	116 (100)	- -

Age (years), (Mean ± SD)	24.0 ± 10.99	18.4 ± 9.66	- -	- -

Number of transfused units, (Mean ± SD)	53.3 ± 22.2	27.7 ± 17.6	29.7 ± 19.1	0.019 (S)

Sex, n (%):				0.73 (NS)
Male	4 (6.3)	59 (93.7)	63 (100)	
Female	5 (9.4)	48 (90.6)	53 (100)	

Age distribution in years, n (%):				0.001 (S)
Child (< 14)	1 (1.4%)	72 (98.6%)	73 (100%)	
Adult (> 14)	8 (18.6)	35 (81.4)	43 (100%)	

Type of hemoglobin, n (%):				0.73 (NS)
HbSS	4 (6.45)	58 (93.55)	62 (100)	
S/B-thal	5 (9.26)	49 (90.74)	54 (100)	

Splenomegaly, n (%):				0.009 (S)
Yes	7 (28)	18 (72)	25 (100)	
No	2 (2.2)	89 (97.8)	91 (100)	

**Table 2:** Alloantibody specificity and frequency in 116 Palestinian SCD patients.

**Alloantibody specificity**	**Frequency and percentage of alloantibody, n (%)**
Anti-K	3 (33.3%)

Anti-E	2 (22.2%)

Anti-D	1 (11.1%)

Anti-C	1 (11.1%)

Anti-c	1 (11.1%)

Anti-D, -C	1 (11.1%)

Total	9 (100%)

**Table 3:** Characteristics of alloimmunized SCD patients (HbSS and HbS/*β*-thal).

**Patient No.**	**Sex**	**Age** **(years)**	**Hemoglobinopathy**	**No. of blood units transfused**	**Alloantibody detected**	**Indication for transfusion**	**Transfusion reaction**
**1**	F	20	HbSS	73	Anti-K	Anemia, Hb< 7g/dl	No

**2**	M	48	HbSS	98	Ant-C + D	Painful crisis, anemia	DHTR^1^, jaundice

**3**	F	31	HbSS	57	Anti-K	Painful crisis, anemia	DHTR, jaundice

**4**	M	24	HbSS	48	Anti-C	Joint pain, painful crisis Anemia	No

**5**	M	8	HbS/ *β*-Thal.	20	Anti-D	Anemia, Hb< 8g/dl	IHTR^2^, jaundice

**6**	F	18	HbS/ *β*-Thal.	42	Anti-E	Anemia, joint pain	No

**7**	F	26	HbS/ *β*-Thal.	51	Anti-K	Anemia, Hb< 6g/dl	Jaundice

**8**	M	22	HbS/ *β*-Thal.	54	Anti-E	Anemia, painful crisis	No

**9**	M	19	HbS/ *β*-Thal.	37	Anti-c	Anemia, painful crisis	Jaundice

^1^DHTR: delayed hemolytic transfusion reactions; ^2^IHTR: immediate hemolytic transfusion reaction.

## Data Availability

The data used to support the findings of this study are available from the corresponding author upon request.
